# Incidence of venous thromboembolism following head and neck surgery

**DOI:** 10.1007/s00405-023-08112-8

**Published:** 2023-07-17

**Authors:** Ida E. Albertsen, Nina M. Lyhne, Torben B. Larsen, Peter B. Nielsen

**Affiliations:** 1 Department of Otolaryngology, Head and Neck Surgery and Audiology, Aalborg University Hospital, Hobrovej 18-22, 9000 Aalborg, Denmark; 2 Aalborg Thrombosis Research Unit, Aalborg University, Aalborg, Denmark; 3 Department of Cardiology, Aalborg University Hospital, Hobrovej 18-22, 9000 Aalborg, Denmark

**Keywords:** Venous thromboembolism, Surgery, Cancer, Anticoagulation

## Abstract

**Purpose:**

Venous thromboembolism (VTE) is associated with significant morbidity and mortality in patients undergoing surgery, but conflicting data exist on VTE risk in patients undergoing head and neck surgery for malignant and non-malignant conditions. Our aim was to examine the risk of VTE among patients with and without cancer undergoing head and neck surgery.

**Methods:**

We conducted a nationwide cohort study to examine the risk of VTE among patients with an otolaryngological diagnosis using data from the Danish National Patient Register between 2010 and 2018. Analyses were stratified by cancer and anatomical areas of the surgical procedure.

**Results:**

In total, 116,953 patients were included of whom 10% (*n* = 12,083) had active cancer. After 3 months, 1.2% of the patients with cancer and 0.3% of the patients without cancer experienced VTE, respectively. For patients undergoing mouth/throat surgery, 0.8% with cancer and 0.2% without cancer had VTE, respectively. After nose/sinuses surgery 0.7% and 0.2%, respectively. No patients experienced VTE after ear surgery; and after endoscopies the numbers were 1.3% and 0.6% respectively.

**Conclusions:**

While the minority of patients undergoing head and neck surgery develop VTE postoperatively, the risk increases among those with cancer. To support clinical decision making on anticoagulation, risk stratification tools could be further developed to recognize this hazard in patients with cancer undergoing head and neck surgery.

**Supplementary Information:**

The online version contains supplementary material available at 10.1007/s00405-023-08112-8.

## Introduction

Venous thromboembolism (VTE), comprising both deep venous thrombosis (DVT) and pulmonary embolism (PE), is a common postoperative complication, especially among those with cancer among [1]. PE, in particular, is recognized as an important cause of potentially preventable death among postoperative patients [2]. Not to be underestimated, DVT is associated with high morbidity and mortality [3, 4] with potential of severe complications of post-thrombotic syndrome and associated high healthcare cost and quality of life burden [5, 6]. Prevention as well as early detection and treatment VTE can reduce the associated morbidity and mortality [7]. However, in the surgical population, especially, the benefit of pharmacological thromboprophylaxis (i.e., anticoagulation) must be weighed against the risk of bleeding.

The frequency of postoperative VTE in the head and neck surgery population varies widely from 0 to 26% [8–11]. Accordingly, assessment of the risks and benefits of thromboprophylactic measures for head and neck surgery patients and determination of guideline recommendations have been challenging. A meta-analysis from 2018 including five studies focused on head and neck patients, all with cancer, described a VTE incidence of 5% for patients undergoing resection [12]. Conversely, in another meta-analysis from 2018 including 29 studies studying patients with and without cancer, an overall VTE risk of 1.5% was observed [13]. Stratification by other relevant factors, such as anatomical location and age, has not performed in the majority of such studies. To support the clinical decision-making on thromboprophylaxis, the American College of Chest Physicians (ACCP) published evidence-based guidelines for the prevention of VTE in surgical patients [7]. The guidelines tailor recommendations to individual surgical specialties and also recommend use of the Caprini risk score for selected surgical specialities [14]. Unfortunately, the ACCP guidelines do not include specific recommendations for head and neck surgery. Numerous obstacles hinder guidelines for prevention of VTE in head and neck surgery: broad heterogeneity of procedure type, variable risk among those undergoing surgery for malignant and non-malignant disease, and lack of specific risk stratification tools in the otolaryngology population.

To provide more clarity regarding the heterogeneous risk of otolaryngology patients, we assessed the frequency of VTE in patients undergoing head and neck surgery, stratified according to cancer status, using contemporary data from a nationwide Danish cohort.

## Materials and methods

This study was an observational cohort study analysing Danish nationwide administrative registry data from year 2010–2018.

### Data sources

The Danish Civil Registration System assigns a unique, central personal registration number (CPR number) to each resident, either at birth or upon immigration, which allows unambiguous individual-level linkage of health registries [15]. This study was based on linkage of three nationwide Danish registries: (1) The Danish National Patient Registry, which has tracked hospitalizations since 1977, and outpatient and emergency department visits at all hospitals in Denmark since 1995 [16]; (2) The National Prescription Register, which records detailed information on purchase date, Anatomical Therapeutic Chemical [ATC] classification code, package size and dosage for every prescription withdrawal in Denmark since 1994 [16]; (3) The Danish Person Registry, which contain data on gender, date of birth, vital and emigration status [17]. All codes used in this study are presented in eTable 1 in the Supplement.

### Study population

The study population consisted of adult patients (age ≥ 18 years) with a first-time head and neck surgery code; the date of the procedure code was defined as the index date. Study subjects were identified from the National Patient Register. We included all in- and outpatients registered with a head and neck surgery diagnosis code from January 2010 through December 2018. ICD-10 codes for surgeries used in this project are listed in eTable 1. Due to overlap with other specialities minor surgical procedures (ICD-10 code ´KQ´, ´KT´, ‘KDCA10’, ‘KDCA20’), surgeries on eye-area (ICD-10 code ´KC´), and certain endoscopies (ICD-10 code ‘KUDQ12’, ‘KUDH02A’, ´KUDB´ and ‘KUDH02’), were not included.

To ensure an adequate retrospective time window for assessment of risk factors in the national registries prior to the surgery, we excluded patients who had not been citizens in Denmark for at least two years prior to surgery. Because of a continued indication for anticoagulation, we also excluded patients with a diagnosis code of atrial fibrillation or heart valve replacement at any time before head and neck surgery, and patients who had claimed a prescription of oral anticoagulant treatment within the past year before the surgical procedure.

### Baseline characteristics and comorbidity

Selected baseline characteristics and comorbidities were identified from the national registries using ICD-10 and ATC codes. Demographic factors, relevant VTE-associated factors, as well as available factors used in the Caprini risk scoring system (eTable 2 in the Supplement), were used to characterize the population [14].

Depending on type of condition and its relevant time-dependent effect on VTE risk, patient characteristics were searched either dating 3 months or 10 years prior to the index event, as done previously [18]. The cohort was stratified according to cancer, which was defined as those who had a record of a cancer diagnosis code within 1 year before the index event. This approach was chosen to ensure that a diagnosis code reflected active cancer (excluding nonmelanoma skin cancer). Patients with a previous cancer diagnosis code were considered as suffering from a history with cancer.

### Outcome

The study outcome was VTE (deep venous thrombosis or pulmonary embolism) occurring within 90 days of a first-time head and neck surgery. To ensure validity of the outcome, hospital discharge diagnosis codes of VTE were required to be given in combination with an imaging examination code. For incident VTE diagnosis codes, this algorithm has been shown to ensure a positive predictive value of 91% [19].

### Population controls

We compared the VTE risk for patients undergoing head and neck surgery with matched controls from the general population. Specifically, we used the Danish Civil Registration System to select 5 population controls for each case, matched for age (within 5 years of the birth year) and sex. We selected controls using risk-set sampling: each control had to be alive and at risk of a first VTE on the index date of the case to whom the subject was matched [20]. Controls were assigned an index date identical to that of each corresponding case.

### Statistics

All included patients were described at the date of surgery according to demographics and baseline comorbidities and were stratified by cancer status. Characteristics were described using proportions for categorical variables and medians with interquartile range for continuous variables.

Patients were followed from the date of the head and neck procedure until VTE, death, emigration, or end of the study, whichever occurred first. Crude incidence rates were calculated as number of events divided by 100 person-years. Cumulative incidence functions by means of the Aalen-Johansen estimator, assuming death as competing risk, were used to estimate VTE risk and to depict the absolute VTE risk during follow-up.

Because previous surgery for patients with suspected VTE is considered as a prognostic factor dating 3 months back, we reported results at 3-month follow-up as the main results [21].

Pharmacological thromboprophylaxis use in hospitalised patients from the Department of Otolaryngology, Head and Neck Surgery and Audiology, Aalborg University Hospital, Aalborg, Denmark during year 2021 was ascertained and presented to provide insights on the prophylactic management of these patients.

### Supplementary and sensitivity analyses

Since some patients might have undergone surgery with a previously unrecognized cancer, we did a supplementary analysis, by restriction of the study population to patients free from cancer at baseline. In this sub-cohort, we applied a time-dependent stratification on patients who did and did not develop cancer during follow-up. Furthermore, to clarify the short-term VTE risk after surgery, the VTE risk was also investigated within 7 days after surgery.

The following additional analyses were conducted: (1) VTE risk stratifying patients in age categories as suggested in the Caprini score [22] (< 41 years, 41–59 years, 60–74 years, > 74 years); and (2) VTE risks after surgery according to varying anatomic areas (surgeries related to mouth/throat, nose/sinuses, ear, and endoscopies, respectively).

Analyses were conducted using Stata/MP version 16 (StataCorp LP). This study was conducted in compliance with the General Data Protection Regulation and is part of North Denmark Region’s record of processing activities (j.no. 2017–68). Other approvals were not necessary according to Danish legislation.

## Results

**Fig. 1:**
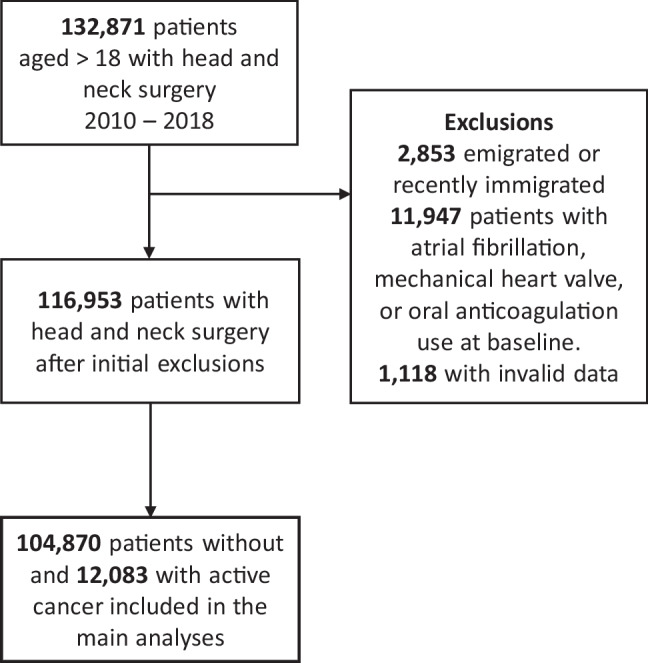
Flowchart of the study population

The study population comprised 116,963 patients who has undergone head and neck surgery between year 2010 to 2018 (Fig. 1). Baseline characteristics are presented in Table 1. Of these, 12,083 patients (10%) had cancer (47.2% female; mean age 66.2 years), and 104,870 patients (90%) did not have cancer (48.6% female; mean age, 51.2 years).

**Table 1:** Descriptive characteristics of 116,953 head and neck surgery patients according to cancer status

Characteristic, no (%)	Cancer, *n* = 12,083 (10%)	No cancer, *n* = 104,870 (90%)
Female sex	5,704 (47.2)	50,947 (48.6)
Age, mean (SD)	66.2 (12.1)	51.2 (19.5)
**Surgery type**		
Operations in relation to the mouth/throat	2,755 (22.8)	43,855 (41.8)
Operations in relation to the nose/sinuses	450 (3.7)	21,973 (21.0)
Operations in relation to the ear	279 (2.3)	13,475 (12.8)
Endoscopies	8,599 (71.2)	25,567 (24.4)
**Persistent risk factors**		
History of VTE	242 (2.0)	1,050 (1.0)
History of cancer	45 (0.4)	1,000 (1.0)
Congestive Heart Failure	844 (7.0)	4,816 (4.6)
Inflammatory bowel disease	135 (1.1)	1,246 (1.2)
Chronic obstructive pulmonary disease	1,274 (10.5)	5,597 (5.3)
Diabetes	881 (7.3)	5,572 (5.3)
Renal disease	399 (3.3)	3,117 (3.0)
Moderate/Severe liver disease	195 (1.6)	1,714 (1.6)
Hypertension	3,988 (33.0)	20,220 (19.3)
Varicose veins	198 (1.6)	1.506 (1.4)
Rheumatic disorder	562 (4.7)	3,930 (3.7)
Obesity	302 (2.5)	2,618 (2.5)
Inherited thrombophilia	n/a	96 (0.1)
Antiphospholipid antibody syndrome	n/a	15 (0.0)
Lupus Erythematosus	13 (0.1)	83 (0.1)
Temporary risk factors		
Other recent major surgery	729 (6.0)	4,244 (4.0)
Immobilization	2,423 (20.1)	5,436 (5.2)
Pregnancy or puerperium	12 (0.1)	286 (0.3)
Central venous catheter	424 (3.5)	3,681 (3.5)
Fracture/trauma	320 (2.6)	10,147 (9.7)
Sepsis	311 (2.6)	1,777 (1.7)
Pneumonia	968 (8.0)	5,391 (5.1)
Stroke	115 (1.0)	783 (0.7)
Cardiac arrest	35 (0.3)	911 (0.9)
Ischemic heart disease	297 (2.5)	1,980 (1.9)
Gastrointestinal bleeding	109 (0.9)	623 (0.6)
Major bleeding	39 (0.3)	172 (0.2)
Hormone replacement	140 (1.2)	8.995 (8.6)

Patients with cancer primarily had an endoscopic procedure performed (71.2% of the patients with cancer vs. 24.4% of the patients without cancer) whereas patients without cancer primarily had surgery on mouth/throat performed (41.8% vs 22.8%, respectively). A greater proportion of cancer patients had hypertension (33.0% vs. 19.3%) and were recently immobilized (20.1% vs. 5.2%). However, a greater proportion of those without cancer had recent trauma or fracture (2.6% with cancer vs 9.7% without cancer). During follow-up, 13.7% of the patients with cancer died compared with 4.0% of patients without cancer.

**Fig. 2:**
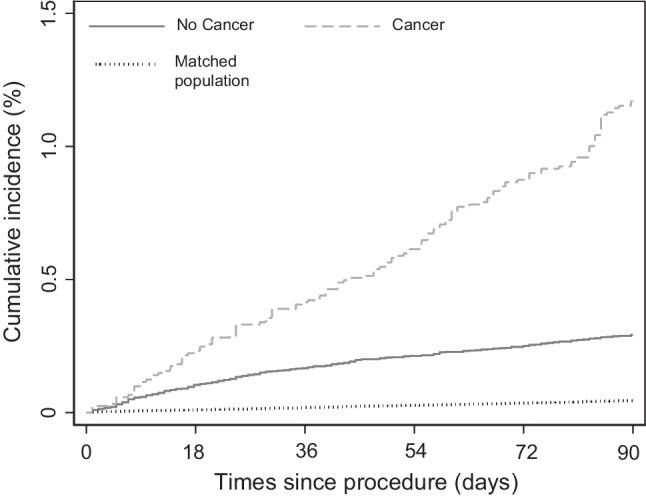
90-days cumulative risk of venous thromboembolism by cancer status and for matched population

A total of 444 patients experienced a VTE event during 3-month follow-up. At 3-month follow-up, VTE rates per 100 person-years were 5.08 for patients with cancer and 1.30 for patients without cancer (Table 2). Figure 2 shows the cumulative incidence proportions of VTE for head and neck patients undergoing surgery and for matched controls. At 3-month follow-up, the absolute VTE risk was 1.2% for patients with cancer 0.3% for those without (Table 2). For the matched controls, a total number of 248 had VTE after 3 months corresponding to a risk of < 0.1%.

**Table 2:** 90-days venous thromboembolism rates per 100 person-years and cumulative risk according to cancer status for 116,953 surgical patients

	Number of events	Rates/100 person-years (95% CI)	Cumulative risk (95%CI)
Cancer	140	5.1 (4.3–6.0)	1.2 (1.0–1.4)
No cancer	304	1.2 (1.1–1.4)	0.3 (0.3–0.3)

During year 2021 on the Department of Otolaryngology, Head and Neck Surgery and Audiology, Aalborg University Hospital, 21 patients out of 1384 hospitalised patients (1.5%) received prophylactic doses of dalteparin or tinzaparin.

### Supplementary and sensitivity analysis

**Fig. 3:**
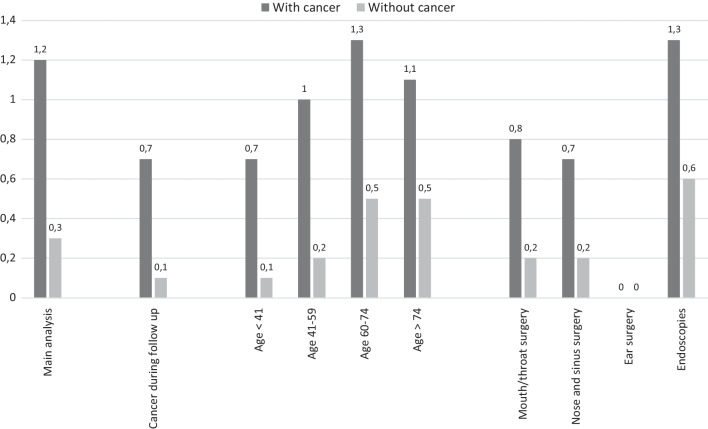
90-days cumulative risk (%) of venous thromboembolism according to selected sub-groups

Results of the supplementary analysis restricted to patients free from cancer at baseline and stratified on development of cancer during follow-up, showed an overall low VTE risk. Specifically, patients developing cancer during follow-up had a VTE risk of 0.7% compared with 0.1% for the patients without cancer (eTable 3). Estimating the short-term VTE risk, 8 patients out of 12,083 with cancer and 52 patients out of 104,870 free from cancer had VTE within 7 days of surgery, both groups with corresponding risk of 0.1% or less. Cumulative risk for the remaining supplementary and sensitivity analyses are shown in Fig. 3 and eTable 4 in the Supplement.

Sensitivity analyses stratifying on age categories showed highest risk for patients with cancer aged 60–74 (1.3%), and lowest risk for patients without cancer aged < 41 years (0.1%). Stratification by anatomical areas demonstrated that VTE was primarily observed in patients with an initial endoscopic procedure (1.3% for patients with cancer vs. 0.6% for patients without cancer). VTE rates for the supplementary analyses are presented in eTable 4 in the Supplement.

## Discussion

In this large nationwide cohort of patients undergoing head and neck surgery, we observed that few patients suffered VTE after head and neck surgery. However, the highest risk of VTE was found among postoperative patients with cancer. Older patients and those undergoing endoscopic head and neck surgery demonstrated higher rates of VTE.

Comparison of studies investigating VTE incidence after head and neck surgery has been challenging due to several epidemiologic differences. First, the follow-up period has varied widely, from VTE evaluated only until discharge to assessment out to 6 weeks after discharge [12, 23]. Second, complexity and length of head and neck surgery can vary greatly [8, 10, 24]. In addition, some studies have chosen to include both symptomatic and asymptomatic VTE while others have focused on only symptomatic events [25]. Some studies include both patients with and without cancers, while others have been confined to patients either with or without malignancy [11, 12, 23]. Statistically, the issue of death as a competing risk for VTE has rarely been considered, e.g. by using the Aalen-Johansen estimator, assuming death as competing risk [26]. Finally, some studies use medical thromboprophylaxis others have relied upon mechanical prophylaxis [27]. These differences from population to methodology have limited the ability to obtain consistent estimates of VTE risk.

A systematic review and meta-analysis including five prospective studies of head and neck cancer patients undergoing tumour resection, found a VTE incidence of 5% [12]. In three of the studies, the follow-up period was not reported, which challenges the interpretation of the estimated 5% risk. In these studies, pharmacological thromboprophylaxis was used routinely only in two of five studies. Hence, the study also concluded that administration of pharmacological thromboprophylaxis was inconsistent in head and neck cancer surgery indicating that current guidelines on thromboprophylaxis in cancer surgery have yet to be implemented in routine clinical practice. In another systematic review and meta-analysis including 23 studies with a total of 618,264 patients including patients with and without cancer, a VTE risk of 0.4% was estimated. Of the included studies, 12 studies evaluated VTE outcomes only until discharge, whereas all the other studies looked at outcomes up to 30 days after discharge, except for a single study evaluating VTE risk up to 6 weeks after discharge [23].

A high VTE risk of 26% was noted 30 days after surgery among 133 patients undergoing oral oncologic surgery with simultaneous reconstruction [9]. All patients had routine postoperative ultrasonography performed to assess for asymptomatic deep venous thrombosis. Of 35 patients with detected VTE, three patients had PE without note of whether these were symptomatic. Of note, surveillance ultrasonography is not routine clinical practice after surgery. Furthermore, the clinical significant and treatment of incidental or asymptomatic VTE is subject to fierce debate [28]. Hence, the clinical implication of this study is uncertain. None of these studies report results stratified on anatomical areas.

However, in the context of these prior studies and our current analysis, it appears that the overall incidence of VTE following head and neck surgery appears lower than that of most other surgical specialties [1]. This has important implications because the major concern regarding pharmacological thromboprophylaxis administration has consistently been bleeding. Bleeding within the neck compartment can be life threatening due to its proximity to the airway. Consequently, the concern of bleeding might contribute to low provider prescription of prophylactic anticoagulation and limited patient adherence to thromboprophylaxis protocols. Furthermore, several studies have showed that the benefit from thromboprophylaxis outweighs the risk of bleeding only in high risk patients [8, 25]. The meta-analysed incidence of bleeding complications was 0.9% [23]. The addition of medical thromboprophylaxis did not result in a significantly lower VTE incidence (odds ratio, 0.86;95% CI 0.48–1.52) but produced a higher risk of bleeding (odds ratio, 3.78; 95% CI 2.20–6.48)).

Some patients undergoing head and neck surgery will undergo re-operation within the following months after a first operation. For anatomical surgery types, the highest risk in our study was found for patients with cancer after an endoscopic procedure (1.3%). For some patients this estimate might reflect a higher risk due to re-operation during follow-up. On the other hand, many patients with cancer may shift to radiation therapy, associated with a relative low VTE risk, after an initial endoscopy instead of receiving more surgery [29]. Either way, an assessment of VTE risk may influence further therapy as well as prophylactic measures. To potentially help inform anticoagulant treatment strategies, future studies may focus on evaluating why VTE occurs and which patients may be specifically at risk of developing VTE.

For surgical patients in general, prognostic factors for VTE include general anaesthesia, prolonged surgical time, postoperative immobilisation, mechanical trauma to venous structures, stasis, and cancer, and reconstructive surgery. Among patients undergoing surgery, surgical duration has been shown to be directly proportional to risk of VTE [30]. For patients with cancer, the 2021 American Society of Hematology guideline recommends pharmacological rather than mechanical thromboprophylaxis for all cancer patients undergoing a surgical procedure at lower bleeding risk [31]. Yet, current practices in VTE prophylaxis for patients with and without cancer vary widely among the otolaryngology community [32, 33]. In a survey of otolaryngologists, 88% indicated that guidelines for the prevention of VTE would be useful [34]. Nevertheless, in a retrospective study evaluating use of thromboprophylaxis in oncologic patients undergoing head and neck surgery, only 568 (56%) out of 1018 received appropriate thromboprophylaxis [8].

Despite efforts in VTE prevention, international guidelines do not provide recommendations specifically for head and neck surgery. The ACCP has made recommendations for some individual surgical specialties, but not for head and neck surgery. For other surgical specialities, the Caprini risk assessment model is recommended [22]. The Caprini score has undergone limited validation in the otolaryngology patient population [25, 35, 36]. In a retrospective validation study of 2016 head and neck patients undergoing surgery, an overall VTE risk of 1.3% after 30 days was observed. The study concluded that only patients with a Caprini score > 8 had a VTE risk higher than 3%. This contrasts with the ACCP recommendations in which only patients with a Caprini score ≥ 3 (3% risk) are recommended to receive medical thromboprophylaxis [7]. Accordingly, in 2022 an adapted model of the Caprini score for otolaryngology patients was published, suggesting a higher score cut-off value (≥ 5) before recommending medical thromboprophylaxis [36]. In our study, patients with cancer aged 60–74 and patients with cancer undergoing an endoscopic procedure exhibited the highest VTE risk (1.3%). As such, the applicability of the Caprini score for patients undergoing head and neck surgery in our cohort may be questionable. To our knowledge, no formal validation study on otolaryngology patients assessing calibration and discriminative power of the Caprini model has been performed [37]. Hence, no international guidelines recommend routine use of the Caprini score for head and neck surgery patients.

## Limitations

Limitations of our study include those typically of large dataset analyses using diagnostic codes. VTE ICD-10 coded records may not reflect an actual VTE event because of inaccurate coding and misclassification. However, our approach minimizes this risk by requiring the VTE diagnosis code to be in combination with relevant imaging examinations ensuring a positive predictive value of 91% for the diagnosis [19]. Yet, given the register-based study type, we were not able to include asymptomatic VTE, and therefore, the true VTE risk is anticipated to be higher [38]. No formal study has been performed on thromboprophylaxis use in Danish otolaryngology patients. Use of thromboprophylaxis during hospitalization in 2021 in Aalborg Department of Otolaryngology was low (1.5%). The department represents the fourth largest otolaryngology department in Denmark. Although not necessarily similar throughout the country, these data underscores that thromboprophylaxis is not routinely prescribed for most head and neck surgery patients.

Some degree of misclassification is expected in the non-cancer group. Our supplementary analysis showed that 5% developed cancer during follow-up. The VTE risk was investigated separately for this group and found lower than the risk for patients with cancer known at baseline.

The ICD codes for VTE do not differentiate in proximal/distal deep vein thrombosis nor in pulmonary embolism location (central/sub-segmental), which may be a limitation of our study. Also, the procedure codes for surgery do not hold any information on the length of the surgery. Finally, the Danish population may not be representative of other populations with greater variability in race and ethnicity, affecting the external validity of our results. The nature of this study was descriptive and causal conclusions cannot be based on the observations.

﻿We followed patients in national registries with prospectively collected data and virtually complete follow-up in a setting with free access to health services, thus largely eliminating selection bias [17].

## Conclusion

The use of medical thromboprophylaxis in otolaryngology is an often-disputed clinical topic. Overall, the minority of patients suffer from VTE after head and neck surgery. However, the risk is higher in those patients with cancer. For patients with cancer, risk stratification could be further developed to help identify patients at highest VTE risk undergoing head and neck surgery in whom the incidence of postoperative VTE might warrant more routine thromboprohylaxis. Patients without cancer have a low VTE risk and may have a risk–benefit ratio that argues against routine prophylactic anticoagulation.

### Supplementary Information

Below is the link to the electronic supplementary material.

Supplementary file1 (DOCX 30 KB)

## Data Availability

Access to data was granted through institutional authorization to the Danish Health Data Authority. Data supporting this study cannot be made available outside the authorization from the Danish Health Data Authority.

## Abbreviations

ATC: Anatomical therapeutic chemical
CI: Confidence interval
ICD: International classification of diseases
VTE: Venous thromboembolism
