# Supplementary material for: Incidence of venous thromboembolism following head and neck surgery
Source: Eur Arch Otorhinolaryngol. 2023 Jul 17;280(11):5081–9. doi: 10.1007/s00405-023-08112-8 (PMC10562290; doi:10.1007/s00405-023-08112-8)
Supplement: Supplementary file 1 — Supplementary file1 (DOCX 30 KB) [file 405_2023_8112_MOESM1_ESM.docx]

***Supplementary material***

**eTable 1: ICD 10 codes and ATC codes used in this project**

**eTable 2: The Caprini score**

**eTable 3: 90-days venous thromboembolism rates per 100 person-years and cumulative risk according to cancer status during follow-up for 104,870 patients free from cancer at baseline**

**eTable 4: 90-days cumulative risk and venous thromboembolism rates per 100 person-years according to cancer status for selected sub-groups.**

**eTable 1: ICD 10 codes and ATC codes used in this project**

|  | **ICD 10 Codes** | **ATC Codes** |
| --- | --- | --- |
| **Venous thromboembolism outcome** | | |
| Deep Venous Thrombosis | I801, I802, I803, I808, I809, I822, I823, I829 |  |
| Pulmonary Embolism | I26 |  |
| Venous thromboembolism | I26, I801, I802, I803, I808, I809, I828, I829, I822, I823 |  |
| **Persistent risk factors (coded up to 10 years before surgery)** | | |
| Cancer (registered within 1 year before incident surgery) | C (non-melanoma skin cancer C44 not included) |  |
| History of VTE | I26, I801, I802, I803, I808, I809, I828, I829, I822, I823 |  |
| Congestive Heart Failure | I50, I110, I130, I132, I420 |  |
| Inflammatory bowel disease | K50, K51 |  |
| Chronic obstructive pulmonary disease | J40, J41, J42, J43 J44, J961 |  |
| Diabetes | E10, E11, E14 |  |
| Renal disease | N03, I12, I13, N11, N14, N15, N16, Q61.1-61.4, N18, N19, N26, N27, N07 |  |
| Moderate/Severe liver disease | B150, B160, B162, B190, K704, K72, K766, I85 |  |
| Hypertension: We identified subjects with hypertension from combination treatment with at least two of the following classes of antihypertensive drugs. |  | I· Alpha adrenergic blockers (C02A, C02B, C02C)  II· Non-loop diuretics (C02DA, C02L, C03A, C03B, C03D, C03E, C03X, C07C, C07D, C08G, C09BA, C09DA, C09XA52)  III· Vasodilators (C02DB, C02DD, C02DG, C04, C05)  IV· Beta blockers (C07)  V· Calcium channel blockers (C07F, C08, C09BB, C09DB)  VI· Renin-angiotensin system inhibitors (C09) |
| Varicose veins | I83 |  |
| Rheumatic disorder | M05-14, M46, M47 |  |
| Atrial Fibrillation or Flutter | I48 | Excluded |
| Mechanical Heart Valve | Z952 Z953 Z954 |  |
| Obesity | E660B-H,  BQFT03 |  |
| Inherited thrombophilia | D685 |  |
| Antiphospholipid antibody syndrome | D686 |  |
| Lupus Erythematosus | L93 |  |
| **Temporary risk factors (coded 3 months before surgery)** | | |
| Other recent major surgery | KA, KF, KH, KK, KL, KM, KN |  |
| Immobilization | Hospitalization >/= 3 days |  |
| Pregnancy or puerperium | Z33, O00-O99, excl: O882, O223, O229, O871, O879, O225, O873 |  |
| Central venous catheter | BMBZ61, BMBZ71, BMBZ51, BMLA01-03 |  |
| Fracture/trauma | S00-T14 |  |
| Sepsis | A40, A41 |  |
| Pneumonia | J12, J13, J14, J15, J16, J17, J18 |  |
| Stroke | I60, I61, I62, I63, I64 |  |
| Cardiac arrest | I46 |  |
| Ischemic heart disease | I20 I21 I22 I23 I24 I25 |  |
| Gastrointestinal bleeding | K250 K252 K254 K260 K262 K264 K270 K272 K274 K280 K282 K290 K920 K921 K922 |  |
| Major bleeding | D62 J942 H113 H356 H431 N02 R04 R31 R58 |  |
| **Medicine (prescription claims 1 year before incident VTE)** | | |
| Statins |  | C10 |
| Clopidogrel |  | B01AC04 |
| Aspirin |  | B01AC06 |
| Apixaban |  | B01AF02 |
| Rivaroxaban |  | B01AF01 |
| Edoxaban |  | B01AF03 |
| ﻿Dabigatran |  | B01AE07 |
| Warfarin |  | B01AA03 |
| ﻿Phenprocoumon |  | B01AA04 |
| Hormone replacement |  | G03A, G03C, G03F |
| **Imaging Examinations (used to validate recurrent VTE)** | | |
| Ultrasonography UE | UXUG |  |
| MR venography | UXZ52 |  |
| Angiography | UXAG, UXAC10 |  |
| Ventilation-perfusion examination | WLHGS |  |
| CT-scan | UXCG, UXCC |  |
| Flebografi | *UXAG05* |  |
| **Surgery after start follow-up** | | |
| Bleeding after surgery | DT810 |  |
| **Surgery type** | | |
| **Operations on mouth/throat** | | |
| Operations on the thyroid gland | KBA |  |
| Operations on the parathyroid gland | KBB |  |
| Operations on glomus caroticum | KBD |  |
| Operations on larynx | KDQ |  |
| Operations on the palate | KEHA KEHB |  |
| Operations on tongue and lower mouth | KEJ |  |
| Operations on cheek | KEK |  |
| Operations on salivary glands | KEL |  |
| Operations on tonsils and adenoid tissue | KEM |  |
| Operations on throat and surrounding soft tissue | KEN |  |
| Tracheotomies, removal of pathological tissue and operations on lesions | KGBA |  |
| Tracheostomies and additional operations | KGBB |  |
| Laparoscopic or thoracoscopic excision of pathological tissue in oesophagus | KJCA01 |  |
| Excisions of lymph nodes | KPJD41 KPJD51 |  |
| **Operations in relation to the nose and sinuses** | | |
| Operations on the nose | KDH |  |
| Operations on the nasal septum | KDJ |  |
| Operations related to epistaxis | KDK |  |
| Rhinoplastic operations | KDL |  |
| Operations on sinus maxillaris | KDM |  |
| Operations on sinus ethmoidales and os ethmoidale | KDN |  |
| Operations on sinus frontalis and sinus sphenoidalis | KDP |  |
| **Operations in relation to the ear** | | |
| Operations on the external ear | KDA |  |
| Operations on the ear canal | KDB |  |
| Operations on the eardrum and middle ear | KDC: excluding KDCA10, KDCA20 |  |
| Operations on the small ear bones | KDD |  |
| Operations on processus mastoideus and os temporale | KDE |  |
| Operations on the inner ear | KDF |  |
| Operations on tubauditive | KDG |  |
| **Endoscopies** | | |
| Endoscopies of the ear, nose, and larynx | KUDH KUDM KUDQ, excluding: KUDQ12, KUDH02, KUDH02A, KUDB |  |
| Endoscopies of mouth and larynx | KUE |  |
| Endoscopy of trachea, bronchi, and lungs | KUG |  |
| Tracheoscopy | KUGB |  |
| Bronchoscopy | KUGC12 KUGC02 |  |
| Esophagoscopy | KUJC02 |  |

**eTable 2: The Caprini score**

| **1-Point Risk Factors, All Patients** |
| --- |
| Age 41 to 60 years  *Minor surgery planned*  Recent major surgery (<1 month)  Varicose veins  History of inflammatory bowel disease  *Swollen legs (currently)*  Obesity (BMI >25)  Acute myocardial infarction  Congestive heart failure (<1 month)  Sepsis (<1 month)  Serious lung disease, including pneumonia (<1 month)  Abnormal pulmonary function  Medical patient currently prescribed bed rest |
| **1-Point Risk Factors, Women Only** |
| Oral contraceptives or hormone therapy  Pregnancy or postpartum status (<1 month)  *History of unexplained stillbirth or recurrent spontaneous abortion* |
| **2-Point Risk Factors** |
| Age 60 to 74 years  *Arthroscopic surgery*  Malignant condition (currently or previously)  *Major surgery (>45 minutes)*  Patient confined to bed (>72 hours)  *Immobilizing plaster cast (<1 month)*  Central venous access |
| **3-Point Risk Factors** |
| Age >75 years  History of DVT/PE  *Family history of thrombosis*  Positive factor V Leiden  Positive prothrombin 20210A  *Elevated serum homocysteine level*  Positive lupus anticoagulant  Elevated anticardiolipin antibodies  *Heparin-induced thrombocytopenia*  Other congenital or acquired thrombophilia |
| Factors in italics were not available in the registries.  Abbreviations: BMI, body mass index (calculated as weight in kilograms divided by height in meters squared); DVT, deep venous thrombosis; PE, pulmonary embolism. |

**eTable 3: 90-days venous thromboembolism rates per 100 person-years and cumulative risk according to cancer status during follow-up for 104,870 patients free from cancer at baseline**

|  | **N** | **Number of events** | **Rates/100 person-years (95% CI)** | **Cumulative risk (95% CI)** |
| --- | --- | --- | --- | --- |
| **Cancer status during follow-up** | | | | |
| No cancer during follow-up | 99,386 | 228 | 0.95 (0.83; 1.08) | 0.1 (0.1-0.1) |
| Cancer during follow-up | 5,484 | 76 | 6.01 (4.80; 7.52) | 0.7 (0.6-0.8) |

**eTable 4: 90-days cumulative risk and venous thromboembolism rates per 100 person-years according to cancer status for selected sub-groups.**

|  | **N** | **Number of events** | **Cumulative risk (95% CI)** | | **Rates/100 person-years (95% CI)** | |
| --- | --- | --- | --- | --- | --- | --- |
|  |  |  | **With cancer** | **Without cancer** | **With cancer** | **Without cancer** |
| **Age** | | | | |  |  |
| <41 years | 34,356 | 22 | 0.7 (0.2-1.8) | 0.1 (0.0-0.1) | 2.74 (0.88; 8.49) | 0.30 (0.20; 0.44) |
| 41-59 years | 32,617 | 92 | 1.0 (0.7-1.5) | 0.2 (0.2-0.3) | 4.23 (2.83; 6.31) | 0.93 (0.73; 1.18) |
| 60-74 years | 32,425 | 212 | 1.3 (1.1-1.7) | 0.5 (0.4-0.6) | 5.82 (4.66; 7.28) | 2.13 (1.80; 2.53) |
| >74 years | 17,555 | 112 | 1.1 (0.8-1.5) | 0.5 (0.4-0.7) | 4.76 (3.44; 6.61) | 2.35 (1.87; 2.94) |
| **Anatomical region of surgery** | | | | |  |  |
| Operations in relation to the mouth/throat | 46,610 | 127 | 0.8 (0.5-1.2) | 0.2 (0.2-0.3) | 3.53 (2.33; 5.37) | 0.97 (0.80; 1.18) |
| Operations in relation to the nose and sinuses | 22,423 | 39 | 0.7 (0.2-1.9) | 0.2 (0.1-0.2) | 2.95 (0.95; 9.16) | 0.65 (0.47; 0.90) |
| Operations in relation to the ear | 13,754 | 5 | 0.0 (0.0-0.0) | 0.0 (0.0-0.1) | 0.00 (.-;.) | 0.15 (0.06; 0.36) |
| Endoscopies | 34,166 | 279 | 1.3 (1.1-1.6) | 0.6 (0.5-0.7) | 5.86 (4.88; 7.04) | 2.68 (2.29; 3.12) |
